# Restoring B cell intrinsic tolerance to lupus autoimmunity: a rational strategy for lupus treatment

**DOI:** 10.3389/fimmu.2026.1728403

**Published:** 2026-02-11

**Authors:** Shreya Desikan Shai, Sailee Vijay Chavan, Elaine Zi Huan, Peter M. Kalin, Miriam Feuerman, Christopher A. J. Roman, Chongmin Huan

**Affiliations:** 1 State University of New York (SUNY) Downstate Health Sciences University, Program in Molecular and Cellular Biology, The School of Graduate Studies, Brooklyn, NY, United States; 2 Department of Cell Biology, SUNY Downstate Health Sciences University, Brooklyn, NY, United States

**Keywords:** autoreactive B cells, B cell tolerance, germinal center, lupus treatment, SLE, SMS2/PKCδ tolerance pathway

## Abstract

Systemic Lupus Erythematosus (SLE or lupus) is a chronic autoimmune disease characterized and driven by anti-nuclear antibodies, most prominently anti-double-stranded (ds) DNA IgGs. Lupus remains a serious clinical challenge largely because available medications lack specificity to lupus autoimmunity, unavoidably impairing normal immune responses and often harming other important body functions. Reinforcement of immune tolerance has a great potential for selective suppression of lupus autoimmunity. However, despite extensive efforts, satisfactory clinical outcomes have not yet been achieved. Here, we review relevant literature on the approaches that reinforce immune tolerance for lupus treatment and classify them into following categories based on their mechanisms of action: (1) directly blocking anti-dsDNA antibodies, (2) leveraging regulatory T cell-regulated B cell extrinsic tolerance, and (3) reinforcing B cell intrinsic tolerance. B cell intrinsic tolerance contains various “built-in” self-protective mechanisms that prevent B cell autoimmunity. In lupus patients, impaired B cell intrinsic tolerance to lupus autoimmunity is a root cause of disease development. However, due to the poorly understood tolerance mechanisms, few publications have studied therapeutic approaches that restore B cell intrinsic tolerance to lupus autoimmunity for physiological suppression of disease. To facilitate the development of such approaches, this review concludes by emphasizing a discussion of B cell intrinsic tolerance to lupus autoimmunity, including our recent finding of a B cell intrinsic tolerance mechanism that was required to prevent lupus pathogenesis and could be pharmacologically restored to selectively suppress lupus autoimmunity in a preclinical model.

## Introduction

Systemic Lupus Erythematosus (SLE or lupus) is a chronic systemic autoimmune disease that affects approximately 3.4 million people worldwide, of which ninety percent are women, with women of non-European ancestry being most frequently and severely affected (1, 2). Patients with lupus present with a wide range severity in levels of symptoms including a variety of constitutional, musculoskeletal, renal, neuropsychiatric and dermatologic manifestations (2). Their mortality rate is two-or-three times higher than that of the general population (3). The majority of lupus cases are sporadic, and the exact causes of lupus remain elusive and a subject of intense investigation. However, it is thought that genetic factors may predispose individuals to lupus, and that environmental factors such as exposures to UV light, cigarette smoking, infection with Epstein-Barr virus, certain medications, and social determinants that impact quality and access to health care can all trigger and exacerbate the disease (1–7).

Lupus is mainly mediated by autoantibodies. More specifically, it is characterized and driven by autoantibodies against nuclear components, most prominently anti-double-stranded (ds) DNA IgGs, which are highly specific for the diagnosis of lupus (8). These autoantibodies appear in patients’ circulation before the clinical onset of lupus disease (9). They promote inflammatory injuries in patients mainly through the formation of immune complexes with nuclear antigens, which become available to bind antinuclear antibodies due to excessive cell death, substantial release of neutrophil extracellular traps, or insufficient clearance of extracellular nuclear materials (10–12). These pathogenic immune complexes stimulate innate immune cells, most prominently dendritic cells, to secrete pro-inflammatory cytokines such as type I interferons that are characteristic of this disease. Furthermore, they can cause organ damage, such as nephritis, by depositing in the tissue and activating the complement system. Therefore, targeting lupus autoimmunity by blocking autoantibody production or autoimmune complex formation is essential for effective suppression of lupus disease activity.

Lupus is currently incurable, and lifelong autoimmune suppression is required to control symptoms and prevent flares, which otherwise may cause disability and severe life-threatening complications (13–15). However, no available medications have yet been successful in selectively suppressing lupus autoimmunity. Current lupus medications include steroids, nonsteroidal anti-inflammatory drugs (NSAIDs), disease modifying antirheumatic drugs (DMARDs) such as antimalarial agents and biologics, and chimeric antigen receptor (CAR) T cell therapy. Both steroids and NSAIDs inhibit inflammatory responses through general immune suppression, which unavoidably impairs normal immune functions. In addition, they have many other toxicities, such as gastrointestinal irritation, hepatic toxicity, hypertension, weight gain, osteoporosis, etc. (16, 17) Hydroxychloroquine (HCQ) is an antimalarial agent that has been used as the first-line treatment for lupus for about 70 years. HCQ can reduce lupus activity without significantly increasing the risk of infection or malignancy (18). Although the mechanism of action remains incompletely understood, it is thought that HCQ mainly works by inhibiting the signaling of Toll-like receptors 7 and 9 (TLR7 and TLR9) and cytokine production in immune cells (19). However, HCQ also has multiple off-target adverse effects including nausea, vomiting, diarrhea, and more severe complications, such as retinopathy and cardiotoxicity, which limit its use (18–20). Belimumab and Rituximab are biologics that have a general inhibitory and depletory effect on B cells, which impairs non-autoreactive B cell-mediated immune functions (21, 22). Similarly, recently developed anti-CD19 CAR T-cell therapy also non-selectively depletes B cells (23, 24). Therefore, the long-term standard of care for lupus inevitably harms normal immunity and other body functions, which may force reductions in dosage and duration (25–28). As a result, insufficient suppression of lupus autoimmunity causes frequent flares.

Immune tolerance selectively protects against autoimmunity. To improve clinical outcomes of lupus disease, different strategies are being developed to reinforce immune tolerance to lupus autoimmunity. As shown in **Table 1**, we classify them into following three categories based on their mechanisms: (1) directly blocking anti-dsDNA antibodies, (2) leveraging regulatory T cell (Treg)-regulated B cell extrinsic tolerance, and (3) reinforcing/restoring B cell intrinsic tolerance. The approaches in category (3) are further divided into two groups based on whether the targeted B cell intrinsic tolerance mechanisms have a known physiological role in suppressing lupus autoimmunity. Notably, the approaches of targeting B cell intrinsic tolerance to lupus autoimmunity are least studied due to lack of knowledge of underlying mechanisms. However, this physiological strategy appears feasible and exhibits great potential for improving lupus treatment.

**Table 1:** Published approaches to selective suppression of lupus autoimmunity.

Type of approach	Target	Reagent/Medicine		Reference	Mouse/Human	Relevant findings
Directly blocking anti-dsDNA antibodies	Anti-dsDNA antibodies	DWEYS peptide		Gaynor et al. (29), 1997	Mouse	Prevented the glomerular deposition of R4A, an anti-dsDNA monoclonal antibody, in SCID mice
				Huerta et al. (30), 2006	Mouse	Protected neurons from the toxic effects of autoantibody exposure in BALB/c female mice immunized with MAP.
		DWEYSVWLSN peptide		Putterman et al. (32), 1998	Mouse	Immunization with the peptide induced the generation of autoantibodies including anti-dsDNA IgG, anti-Histone IgG and anti-Cardiolipin IgG and renal immunoglobulin deposition in BALB/c mice.
		FISLE-412		Bloom et al. (33), 2011	Mouse	Suppressed pathogenic activities of lupus patients’ autoantibodies *in vitro* and inhibited the neurotoxicity of anti-dsDNA antibodies in C57BL/6 mice.
		Analogues of FISLE-412		He et al. (34), 2017	Mouse kidney tissue	Suppressed anti-dsDNA antibody binding in the mouse kidney ex vivo.
		AWL		Xia et al. (36), 2016	rat glomeruli	Suppressed anti-dsDNA antibody binding in the rat glomeruli ex vivo.
				Wang et al. (37), 2019	Mouse	Decreased renal IgG deposition and relevant damage in MRL/lpr mice.
Leveraging Treg-regulated B cell extrinsic tolerance	Tregs	IL-2		Humrich et al. (65), 2010 Rose et al. (66), 2019	Mouse	IL-2 induced expansion of Tregs was associated with improved disease activities in NZBWF1 mice.
				La Cava A (60)., 2023 (Review) Akkur et al. (61), 2025 (Review)	Mouse and Human	Multiple mouse and human studies have proved beneficial effects of low-dose IL-2, which reduced disease activity in lupus patients associated with moderately increased Tregs.
				He et al. (68), 2020 Humrich et al. (69), 2022	Human	Failed to meet the primary endpoint in two randomized, double-blind, placebo-controlled clinical trials.
		Rezpegaldesleukin (PEGylated conjugate of IL-2)		Kaufman et al. (70), 2023	Human	Failed to reduce disease activity in a clinical trial.
		IL-33		Mohd Jaya et al. (73), 2020	Mouse	Reduced autoantibodies and attenuated lupus development in young NZBWF1 mice.
		IL-33 blocking antibody		Li et al. (74), 2014	Mouse	Increased Tregs and suppressed lupus activity in MRL/lpr mice
		IL-6 inhibitor		Tackey et al. (78), 2004 (Review)	Mouse and Human	Multiple studies showed that IL-6 blockade prevented increases in anti-dsDNA levels and progression of proteinuria, and improved survival in lupus prone mice. Blocking IL-6 also decreased ex vivo production of human anti-dsDNA antibodies.
				Rovin et al. (79), 2016 Wallace et al. (80), 2017	Human	Failed to meet the primary endpoint in two randomized, double-blind, and placebo-controlled clinical trials, disproving the benefits of using IL-6 blocking antibodies for treating lupus.
		mTOR pathway inhibitor	Rapamycin	Strauss et al. (82), 2009	Human cells	Efficiently expanded human Tregs *in vitro*.
				Warner et al. (83), 1994	Mouse	Prolonged survival and inhibited pathogenesis in MRL/lpr mice.
				Fernandez et al. (84), 2006	Human	Reduced disease activity in lupus patients
			KN-93	Koga et al. (87), 2014	Mouse	Promoted generation and function of Tregs in MRL/lpr mice
				Otomo et al. (88), 2015	Mouse	Mitigated disease in MRL/lpr mice.
			miR-125a	Zhang et al. (89), 2020	Mouse	Nanoparticles loaded with miR-125a could increase Tregs and alleviate disease in MRL/lpr mice.
		pCons		Hahn et al. (90), 2001 Yu et al. (92), 2012	Mouse	Promoted the generation of autoantigen-specific Tregs in association with reduced anti-dsDNA antibodies and relieved pathogenesis in NZBWF1 mice.
				Hahn et al. (93), 2008	Human cells	Caused the expansion of Tregs in cultured peripheral blood mononuclear cells collected from lupus patients.
		hCDR1 (Edratide)		Sharabi et al. (97), 2006	Mouse	Adoptive transfer of hCDR1-treated cells and hCDR1-treated cells enriched with CD4+CD25+ cells significantly reduced proteinuria and glomerular immune complex deposits in NZBWF1 mice.
				Urowitz et al. (98), 2015	Human	The primary endpoints based on SLEDAI-2K and AMS were not met in a clinical trial.
		Nucleosomal histone peptides		Kang et al. (99), 2005	Mouse	Reduced autoantibody, delayed nephritis and prolonged life span in lupus prone (SWR x NZB) F1 mice. Adoptive transfer of Tregs induced by nucleosomal histone peptides had similar beneficial effects in mice.
				Zhang et al. (101), 2013	Human	Transfer of Tregs induced by nucleosomal histone peptides suppressed lupus pathogenesis in patients.
		Other histone-derived peptides		Wu et al. (102), 2002 Wu et al. (103), 2004 Shapira et al. (104), 2011	Mouse	Had similar Treg-associated beneficial effects in lupus-prone mice, such as suppressing the production of autoantibodies and reducing the severity of glomerulonephritis.
Reinforcing B cell intrinsic tolerance by leveraging the inhibitory mechanisms with unknown relevance to lupus	Anti-dsDNA B cells	dsDNA/anti-dsDNA antibody complex		Lebrun et al. (154), 1994 Burny et al. (155), 1997	Mouse	Extended survival, decreased anti-dsDNA antibody production and reduced severity of nephritis in MRL/lpr mice.
		LJP-394 (Abetimus sodium)		Horowitz et al. (157), 2009 (Review)	Mouse and Human	Both mouse and human studies showed that LJP-394 reduced the titers of anti-dsDNA antibodies and the numbers of anti-dsDNA B cells.
				Mosca et al. (158), 2007 (Review)	Human	Multiple studies showed that LJP could reduce anti-dsDNA antibodies and disease activities in lupus patients.
				Furie et al. (159), 2006 Cardiel et al. (160), 2008	Human	Failed to demonstrate statistically significant prolongations in time to renal flare. Thus, failed to meet its primary endpoint in two pivotal clinical trials.
Restoring B cell intrinsic tolerance to lupus autoimmunity	FcγRIIb	DNA-mimicking peptide/anti-FcγRIIb antibody complex		Tchorbanov et al. (175), 2007	Mouse	Selectively silenced DNA-specific B cells and delayed lupus activity in MRL/lpr mice
		Histone 1 peptide/anti-FcγRIIb antibody complex		Mihaylova et al. (177), 2008	Mouse	Reduced anti-Histone 1 antibodies, albuminuria, and the size of lymphoid organs and prevented skin lesions in MRL/lpr mice.
	SMS2/PKCδ pathway	2OHOA		Ou et al. (180), 2021	Mouse	Significantly reduced anti-dsDNA antibodies and relieved lupus pathogenesis without the reduction of total IgGs in NZBWF1 mice.
		BAFF-receptor-Ig, Belimumab		Jackson et al. (236), 2019 (Review)	Mouse and Human	BAFF inhibition with BAFF-receptor-Ig in lupus-prone mice and with Belimumab in patients exhibited a similar modest and apparent effect on the antigen-activated autoreactive B cell repertoire.
		Belimumab		Huang et al. (230), 2018	Human	Patients received a long-term belimumab treatment had a modest reduction in the frequency of activated autoreactive B cells, but not inactive autoreactive B cells, suggesting the possible involvement of overexpressed BAFF in inhibiting GC B cell tolerance.

## Directly blocking anti-dsDNA antibodies

Given that anti-dsDNA antibodies are the key driving force in lupus pathogenesis (8), directly blocking them was logical and one of the first strategies explored for new treatment options. By screening a phage peptide display library, B. Diamond’s group pioneered this approach and identified DWEYS, a peptide that is recognized by R4A, an anti-dsDNA monoclonal antibody with known renal pathogenicity. This DWEYS peptide inhibited R4A’s DNA binding and prevented the deposition of R4A in mouse glomeruli (29). In addition, DWEYS protects neurons from the toxic effects of antibody exposure (30). Further ex vivo studies showed that DWEYS could inhibit lupus patients’ anti-dsDNA antibodies from binding to DNA, supporting its therapeutic potential (31). However, despite the encouraging results, immunization with a similar R4A-specific peptide DWEYSVWLSN induced the generation of autoantibodies and renal immunoglobulin deposition in mice (32), raising concerns that this treatment strategy posed the risk of further enhancement of autoimmunity.

Later, FISLE-412 was developed as a small molecule peptidomimetic based on the structural features of DWEYS that neutralizes anti-dsDNA/NMDAR, but with more stability, oral availability and less immunogenicity than its precursor (33). Like DWEYS, FISLE-412 was able to block patient anti-dsDNA antibodies *in vitro* and ex vivo, as well as inhibit markers of lupus pathogenesis in mice (34, 35). Similarly, ALW (ALWPPNLHAWVP) is another such peptide that inhibits anti-dsDNA antibodies (36). ALW was developed with a screen against a panel of murine IgG anti-DNA antibodies of different isotypes with varied renal pathogenicity. ALW inhibited the binding of anti-dsDNA antibodies to mesangial cells and glomeruli *in vitro* and ameliorated lupus nephritis in the MRL/lpr mouse (37). Though FISLE-412 and ALW showed more promise over previous peptide based approaches (38), effective translation into clinical use has yet to be reported, possibly due to the concern of potential immunogenicity.

## Leveraging Treg-regulated B cell extrinsic tolerance

B cell extrinsic tolerance consists of two main avenues that may prevent the activation of autoreactive B cells: (1) the absence of T cell help from antigen-cognate CD4+ helper T cells due to elimination of autoreactive T cells through T cell tolerance, and (2) the suppression of autoreactive B cells by Tregs (39–42). T cells play important roles in lupus pathogenesis as anti-nucleosome T cells seen in lupus have been hypothesized to aid the generation of antibodies that target nuclear antigens like dsDNA (43, 44), and that some T cell inhibitors showed encouraging results in clinical studies (45). However, effective CD4+ helper T cell-specific targeted therapy has yet to be reported, and several clinical trials targeting T cell-dependent pathways, such as blocking co-stimulatory pathways, did not meet their endpoints (46–48).

In contrast, dysregulated Tregs have been considered to be an important factor contributing to insufficient peripheral B cell tolerance in lupus (49). Tregs have the ability to induce peripheral tolerance to self-antigens in lupus by releasing anti-inflammatory cytokines, such as IL-10 and TGF-β, or by suppressing autoreactive B cells via direct interaction (49–51). Therefore, leveraging Treg-regulated B cell extrinsic tolerance to suppress lupus autoimmunity has been actively pursued as a targeted therapy (52–54). Studies have shown that the transfer of Tregs was effective in the suppression of disease progression in lupus-prone NZBWF1 mice (55–57), as well as in a humanized mouse model of lupus (58), justifying the pursuit of a potential therapeutic intervention. Interestingly, the transfer of self-antigen specific Tregs seemed to have higher specificity and activity to suppress self-antigen-induced pro-inflammatory responses (59). However, as the humanized mouse model used in this study did not support the survival of transplanted B cells, whether self-antigen specific Tregs would have any beneficial effects on B cell tolerance is unknown.

In addition to the transfer of Tregs, bolstering or restoring Tregs with IL-2 has been extensively investigated for treating lupus (60, 61). IL-2 is critical for expanding and maintaining CD25+ Treg populations (62); defective IL-2 signaling in mice results in reduced Treg populations, contributing to several autoimmune phenotypes (63, 64). IL-2 treatment prevented the decrease of Tregs in lupus-prone mice (54), and IL-2 induced Treg expansion was associated with improved disease activities (65, 66). Lymphocytes from lupus patients had decreased production and responsiveness to IL-2 in culture (67). Multiple clinical studies originally showed therapeutic effects of low-dose IL-2, which reduced disease activity in association with moderately increased Tregs (60, 61). Given that therapeutic efficacy of low-dose IL-2 was possibly linked to the inhibition of Tfh and Th17 cells, the contribution of IL-2 to each of these cell populations in relation to the overall outcomes remains to be defined. Despite the positive results achieved by early clinical studies, both single-center and multicenter, randomized, double-blind, placebo-controlled clinical trials failed to meet the primary endpoints (68, 69). Similarly, a recent clinical trial of Rezpegaldesleukin, a PEGylated conjugate of recombinant human IL-2, failed to reduce disease activity in lupus patients (70). These compelling results challenged the effectiveness of targeting IL-2-regulated Tregs for lupus treatment. In addition, they indicate that insufficient IL-2 alone does not drive lupus pathogenesis, which is also supported by the fact that only half (54.8%) of lupus patients had low levels of serum IL-2, compared to 11% in the healthy individuals (71).

Other cytokines that regulate Treg activity have also been explored as targets for lupus treatment. Like IL-2, IL-33 also expands Tregs both *in vivo* and *in vitro* (72). IL-33 administration reduced autoantibodies in NZBWF1 mice (73). A caveat to this finding is that IL-33 is a member of the IL-1 family, which is known to promote proinflammatory responses; inhibition of IL-33, but not IL-33 administration, increased Tregs and suppressed lupus activity in MRL/lpr mice (74). In contrast to IL-2 and IL-33, IL-6 is elevated in lupus patients and correlates with disease activity (75). IL-6 inhibits Tregs via upregulating Th17 cells (76). Additionally, IL-6 can induce B cell hyperactivity, autoantibody production and inflammation (77). Indeed, IL-6 blockade reduced disease activity in multiple mouse models of lupus (78). Unfortunately, two randomized, double-blind, and placebo-controlled clinical trials of using IL-6 blocking antibodies for treating lupus in patients did not show benefits (79, 80).

Intracellular pathways also have been targeted to increase Tregs for lupus treatment and have showed positive results. For example, capitalizing on the observations that overactivation of mTORC1 resulted in defective Tregs (81), and that conversely, inhibition of mTORC1 by rapamycin expanded human Tregs *in vitro* (82), studies showed that rapamycin could relieve lupus pathogenesis in MRL/lpr mice and lupus patients (83, 84). Later, a single-arm, open-label, phase 1/2 trial showed that lupus patients receiving rapamycin had a progressive improvement of disease activity associated with reduced production of IgM and IgA antiphospholipid antibodies (85). Although a retrospective study of 4-year rapamycin treatment showed acceptable tolerance and safety (86), unavoidable inhibition of mTOR-regulated normal functions remains a concern. In agreement with the effectiveness of rapamycin, inhibition of CaMK4, an upstream regulator of mTOR, also increased Tregs and mitigated disease in MRL/lpr mice (87, 88). In addition, targeting this intracellular pathway with miR-125a consistently increased Tregs and alleviated disease in MRL/lpr mice (89), supporting the further exploration of this promising approach to treat lupus and other autoimmune diseases.

Interestingly, the peptides derived from anti-DNA antibodies, such as “consensus“ peptide (pCons) and human complementarity-determining region 1 (hCDR1), also promote the generation of Tregs (90, 91). pCons was an artificial sequence whose design was based on a consensus of T cell stimulatory amino acid sequences derived from the VH regions of several anti-DNA IgGs found in NZBWF1 mice. Administration of pCons promoted the generation of Tregs in association with reduced anti-dsDNA antibodies and relieved pathogenesis in lupus-prone mice (90, 92), and caused the expansion of Tregs in cultured lupus patients’ blood cells (93). However, clinical effectiveness of pCons has yet to be reported. hCDR1 has multiple potential mechanisms of action that are thought to benefit lupus treatment (91, 94–96), but its effects on upregulating Tregs and TGF-beta are essential (97). hCDR1 showed clinically significant effects in some patients but failed to meet its primary endpoints in a phase II clinical trial (98). In addition to autoantibody-derived peptides, peptides containing epitopes of nucleosomal histones also promoted Treg responses and relieved lupus nephritis in lupus prone (SWR x NZB) F1 mice (99). The transfer of Tregs induced by these peptides suppressed lupus autoimmunity in lupus patients (100, 101). Other histone-derived peptides showed similar beneficial effects (102–104).

Overall, while animal studies and early-stage clinical studies have demonstrated exciting beneficial effects of leveraging Treg-mediated B cell extrinsic tolerance to treat lupus, definitive clinical evidence supporting such approaches has yet to be achieved. In addition, Treg deficiency does not appear to be a common defect in lupus patients. Although multiple studies showed decreased proportions of Tregs in lupus patients (105–112), one study reported unchanged proportion of Tregs (113), and some even reported increases in that numbers (114–117). Similarly, the quality of Tregs in lupus has been disputed as well (108, 114, 115). Given the disparate evidence, including inconsistent clinical study results, the potential significance of leveraging Treg-regulated B cell extrinsic tolerance for lupus treatment remains uncertain.

## B cell intrinsic tolerance checkpoints

The random nature of recombination of variable (V), diversity (D), and joining (J) (VDJ) segments and somatic hypermutation (SHM) diversifies B cell receptor (BCR) specificities for protecting against a virtually limitless array of pathogens (118), but also generates autoreactive BCRs as a by-product (119–121). However, B cell intrinsic tolerance deletes or deactivates those autoreactive B cells at multiple checkpoints in B cell development to prevent autoimmune disorders (122–125).

In the bone marrow, up to 75% BCRs generated at this stage exhibit reactivity to self-antigens (121, 126). Central B cell tolerance removes the BCRs possessing high-avidity interactions with self-antigens by various mechanisms including: clonal deletion, anergy and receptor editing (127), among which receptor editing seems to play a dominant role (128, 129). In the periphery, BCR signaling-mediated clonal deletion and anergy remove most remaining autoreactive B cells during the development of transitional, marginal zone and follicular B cells (130). Both follicular and marginal zone B cells participate in the GC response (131). Follicular B cells are the primary B cells that undergo SHM to generate BCRs with high-affinity to foreign antigens as well as autoreactive BCRs. Marginal zone B cells are also able to undergo SHM and generate T cell–independent immune responses, including autoimmunity (132). However, SHM-generated autoreactive GC B cells are negatively selected by a stringent but poorly understood mechanism to guarantee the expansion of B cells with high affinity to foreign antigens (123, 133).

Notably, although defects in both central and peripheral B cell tolerance have been found in lupus patients, accumulating evidence supports a central role of loss of GC B cell tolerance in lupus pathogenesis. Studies of lupus patients and animal models showed that most of their autoreactive B cells had extensive SHM, and reversing SHM point mutations removes lupus autoimmunity in the B cells (134–139). These convincing results indicate that lupus autoimmunity is primarily generated by SHM, which highlights an essential role of GC B cell tolerance in preventing lupus disease. However, the mechanisms of GC B cell tolerance and the causes of their insufficiency in lupus patients are poorly understood, impeding the approaches to pharmacologic restoration of lupus patients’ GC B cell tolerance for physiologically suppressing lupus autoimmunity.

Consistent with the critical protective role of GC B cell tolerance in lupus disease, the extrafollicular (EF) responses, which lack stringent B cell tolerance checkpoints, have been recognized as a driver of lupus pathogenesis (140). Precursors of lupus autoreactive B cells are thought to arise within these EF responses. Specifically, IgD-CD27- double-negative (DN) B cells, a characteristic population in lupus, have DN2 (CD11c+CXCR5-) and DN3 (CD11c-CXCR5-) subsets that differentiate in EF responses (141–143). DN2 cells are sensitive to TLR7 signaling, which predisposes mice and humans to lupus autoimmunity (144), and these cells account for the expansion of DN cells in African-American patients with active lupus disease (145). Notably, DN2 cells exhibit strong similarities with CD11c+T-bet+ age-associated B cells (ABCs), which arise from both GC and EF responses. ABCs are also driven by TLR7 signaling and recognized as the precursors of lupus autoreactive B cells (146). Compared to DN2 cells, DN3 cells have been less studied in the context of lupus. However, a recent study showed that the increase of DN3 cells, but not DN2 cells, was tightly associated with lupus disease activity, suggesting a potentially more important role for DN3 cells in the disease (147). Given that EF responses, as well as DN cells, are particularly sensitive to TLR7 signaling (144, 145, 148, 149), blocking TLR7 signaling is a reasonable approach to suppress lupus autoimmunity. In a recent phase II clinical trial (NCT05162586) for lupus treatment, Enpatoran, a selective TLR7/8 inhibitor, did lead to a reduction in inflammatory markers and improve cutaneous manifestations of lupus. However, it did not meet its primary endpoint to dampen systemic effects, suggesting other mediators/pathways are involved. Therefore, further investigations are necessary to fully elucidate the roles of EF responses and DN2/DN3 cells in the context of lupus treatment.

It is also worth mentioning that the exons encoding the variable regions of lupus autoantibodies typically possess a high load of SHM, a hallmark of GC maturation, contrasting with the low level of SHM observed in DN B cells derived from activated naïve B cells (140–142). However, this discrepancy can be potentially reconciled by studies suggesting that GC-derived memory B cells may acquire DN2 cells’ features and transform into DN2-like cells under inflammatory conditions (150, 151). In this scenario, insufficient GC tolerance can indirectly contribute to the emergence of DN2/DN3 cells in EF responses. Therefore, restoring GC B cell tolerance may serve as a potential strategy against DN2/DN3 cells by blocking the initial development of autoreactive memory B cells in the GC.

## Reinforcing B cell intrinsic tolerance to lupus autoimmunity by leveraging inhibitory mechanisms with unknown physiological relevance to lupus

B cell intrinsic tolerance is manifested in various self-protective mechanisms including those with known or unknown physiological relevance to lupus autoimmunity. Although restoring B cell intrinsic tolerance to lupus autoimmunity is considered an ideal strategy for lupus treatment (152), due to its incompletely understood mechanisms, early therapeutic approaches focused on engineering “artificial” B cell intrinsic tolerance to dsDNA by capitalizing on general B cell inhibitory mechanisms with no established physiological connection to human lupus.

For example, based on the observation that the idiotype-induced generation of anti-idiotypic antibodies may in turn reduce the production of antibodies carrying the idiotype (153), P. Lebrun et al. attempted to use a complex composed of dsDNA for targeting anti-dsDNA B cells and an anti-dsDNA antibody to suppress the production of anti-dsDNA antibodies by anti-dsDNA B cells (154, 155). The authors reported that lupus prone MRL/lpr mice treated with this complex had significantly prolonged survival associated with reduced morphological signs of nephritis (154). A later study confirmed that this treatment reduced the production of anti-dsDNA antibodies and the severity of nephritis in MRL/lpr mice compared to control (155). However, the mechanism underlying this approach is unclear, and there are no reports of further clinical studies. Notably, a study showed that antibodies specific for a particular antigen enhanced helper T cell proliferation in response to the stimulation by the same antigen (156), suggesting the possible enhancement of an autoimmune response by this approach, which would not be desirable for treating lupus.

The mechanism of antigen specific elimination of self-reactive B cells is critical in central and peripheral B cell tolerance (127, 130), as it removes BCRs with high affinity for self-antigens if they are engaged without co-stimulatory signals. This general inhibitory mechanism in B cells is not specific for preventing lupus, but it was used as a basis to design tolerogens that would impose B cell intrinsic tolerance to dsDNA. For example, LJP-394, a dsDNA tolerogen, was developed to delete or deactivate anti-dsDNA B cells. LJP-394 consists of four identical 20-mer dsDNA epitopes covalently linked to a small non-immunogenic carrier to avoid inducing autoantibody generation (157). LJP-394 could selectively crosslink anti-dsDNA BCRs to induce anergy or apoptosis in anti-dsDNA B cells, resulting in B cell tolerance given the absence of T cell help. More than ten clinical studies showed that LJP-394 reduced anti-dsDNA antibodies and disease activities (158). However, in pivotal clinical trials, LJP-394 failed to meet its primary endpoint (157, 159, 160). Despite the fact that LJP-394 reduced almost 50% serum anti-dsDNA antibody titers in patients with lupus, it could not delay or reduce the time to renal flare in patients that had high affinity antibodies to its dsDNA epitopes. Therefore, even up to a 50% reduction of anti-dsDNA antibodies could not generate a satisfactory clinical outcome. The cause of LJP-394’s insufficient efficacy is unclear. It is possible that the mechanism of antigen specific tolerance is not fully active or attenuated at the B cell development stage when anti-dsDNA B cells arise in lupus, reducing the efficacy of this approach. However, the encouraging results achieved were a significant advancement towards selective and effective suppression of lupus autoimmunity.

## Reinforcing FcγRIIb-regulated B cell intrinsic tolerance to lupus autoimmunity

FcγRIIb receptor possesses the immunoreceptor tyrosine-based inhibition motif (ITIM), which negatively regulates B cell activation and antibody production by interacting with the BCR ITAM motif (161). FcγRIIb deficient mice have an increased susceptibility to autoimmune diseases including arthritis, Goodpasture’s syndrome and lupus (162–165). Although initial studies of FcγRIIb deficient mice observed a strain-dependent lupus-like phenotype (166), later studies showed that this phenotype was contingent on the involvement of other genes (162, 163). Many studies focused on the tolerance role of FcγRIIb in GC and post GC responses (167–171), and a recent study showed that B cell specific FcγRIIb deficient mice exhibited aberrant marginal zone activation and extrafollicular autoreactive plasma cell responses with increased serum anti-dsDNA IgGs (172). Lupus patients have about 40% reduction of FcγRIIb expression in marginal zone B cells (172), and it is not clear how this reduced expression may affect FcγRIIb-regulated GC B cell tolerance. Nevertheless, FcγRIIb overexpression in B cells suppressed lupus autoimmunity in mice (173, 174), supporting the approach of targeting FcγRIIb in autoreactive B cells for lupus treatment.

FcγRIIb is expressed on many different cell types. To activate FcγRIIb selectively in lupus autoreactive B cells, a complex containing a DNA-mimicking peptide (DWEYSVWLSN) for targeting anti-dsDNA B cells and an anti-FcγRIIb monoclonal antibody for activating FcγRIIb’s inhibitory activity was generated (175). Indeed, in sick MRL/lpr mice treated with this complex, levels of anti-dsDNA antibodies did not increase, and aggravation of lupus glomerulonephritis was delayed. A similar complex composed of anti-FcγRIIb antibody and histone 1 peptide was made to inhibit anti-histone-1 B cells (176). This complex reduced anti-histone-1 antibodies and albuminuria, and prevented the development of skin lesions in MRL/lpr mice (177). In addition to treatment with anti-FcγRIIb antibodies and fusion proteins alone, combined treatment with bortezomib, a cytotoxic agent, showed beneficial effects in a preclinical model (178). Inhibitory complement receptor type 1 (CD35) in anti-dsDNA B cells was also targeted to inhibit anti-dsDNA B cells. A complex containing an anti-CD35 monoclonal antibody and a DNA-mimicking peptide decreased the number of anti-dsDNA antibody producing B cells in cultured lupus patients’ PBMCs (179). However, despite these promising observations, the therapeutic effects of these complexes in lupus prone mice were considered as partial successes, and there were no further clinical studies reported.

## Restoring SMS2/PKCδ-regulated GC B cell intrinsic tolerance to lupus autoimmunity

### The SMS2/PKCδ tolerance pathway prevents lupus pathogenesis but is attenuated in lupus prone mice and lupus patients

We have reported a novel mechanism of GC B cell tolerance that prevents lupus autoimmunity in mice (180). Sphingomyelin synthase 2 (SMS2) and protein kinase C δ (PKCδ) are two key regulators of this B cell intrinsic tolerance mechanism. SMS2 is a plasma membrane enzyme that produces sphingomyelin (SM) and diacylglycerol (DAG) by transferring phosphocholine from phosphatidylcholine onto ceramide (181). We showed that compared to B cells at other stages, GC B cells in the light zone (LZ), where negative selection occurs, have increased SMS2. Notably, more highly upregulated SMS2 was specifically found in anti-dsDNA B cells located in the LZ. SMS2 deficiency resulted in a lupus-like phenotype in C57/BL6 mice, which was associated with significantly increased frequency of anti-dsDNA GC B cells and ABCs, indicating that SMS2 preferentially prevents the survival of GC B cells possessing lupus autoimmunity (180). Mechanistically, SMS2 formed a complex with PKCδ to allow SMS2-derived DAG to activate PKCδ nuclear translocation, which is known to phosphorylate histone 2B and cause subsequent apoptosis in B cells (182). Consistent with these findings, nuclear PKCδ was observed in anti-dsDNA GC B cells of C57/BL6 mice, and SMS2 deficiency preferentially impaired PKCδ nuclear translocation and apoptosis in anti-dsDNA GC B cells (180). Moreover, GC B cell specific PKCδ deficient mice had a similar lupus phenotype with increased frequency of anti-dsDNA GC B cells. This tolerance mechanism is B cell intrinsic: adoptive transfer of SMS2 deficient naïve B cells into mature B cell deficient (μ deficient) mice resulted in increased anti-dsDNA GC B cells, which was not observed when WT naïve B cells were transferred into SMS2 and μ deficient mice (180).

Notably, DAG can be generated by other enzymes in B cells as well. For example, phospholipase C gamma 2 (PLCγ2), a critical component of BCR signaling pathways, converts the plasma membrane phospholipid phosphatidylinositol-4,5-bisphosphate (PIP_2_) into inositol 1,4,5-trisphosphate (IP_3_) and DAG. It was suggested that PLCγ2-derived DAG could activate PKC epsilon (ε) (183), a novel PKC implicated in the pathogenesis of psoriasis (184). However, we found that SMS2 deficient B cells had significantly reduced plasma membrane DAG, indicating that DAG generated by other enzymes cannot compensate for the loss of DAG derived from SMS2. In addition, the specific physical interaction between SMS2 and PKCδ could facilitate the direct transfer of DAG from SMS2 to PKCδ (180), which further explains the requirement of SMS2 for the activation of PKCδ nuclear translocation.

PKCδ nuclear translocation appears to be GC B cell specific since nuclear PKCδ was undetectable in untouched and uncultured naïve B cells (180, 182). However, in addition to GC B cells, PKCδ is also involved in the tolerance regulation at the immature B cell stage in the bone marrow and at the transitional B cell stage in the periphery, though not via its nuclear translocation. In bone marrow B cells and transitional B cells, PKCδ was required to sensitize B cells to negative selection via activating proapoptotic Ca2+-dependent Erk signaling (185, 186). However, no evidence supports that this particular tolerance mechanism is required for preventing lupus autoimmunity. Rather, the SMS2/PKCδ tolerance pathway in the GC is a PKCδ mediated tolerance mechanism that protects against lupus autoimmunity. This is also supported by our evidence that GC B cell specific PKCδ deficient mice exhibit a lupus-like phenotype and that activation of SMS2 restored GC B cell PKCδ nuclear translocation and alleviated lupus pathogenesis in NZBWF1 mice (180).

The significance of the SMS2/PKCδ tolerance pathway in humans was validated by showing that SMS2 expression in lupus patients’ B cells and ABCs are reduced more than 90% (180), and hypomorphic mutations in *PRKCD* cause lupus pathogenesis in patients (187–190). Furthermore, in line with the female biased lupus-like phenotype in NZBWF1 mice (191), we found that female NZBWF1 mice had significantly reduced SMS2 expression in GC B cells compared to the male littermates (SS and CH, unpublished observations), which is associated with impaired PKCδ nuclear translocation in these cells (180). These results suggest that the SMS2/PKCδ tolerance pathway is functionally conserved in humans and mice.

### Potential regulation of the SMS2/PKCδ tolerance pathway

To understand the regulation of the SMS2/PKCδ tolerance pathway, we examined the regulation of B cell SMS2 expression given that the transcription of *SGMS2*, which encodes human SMS2, is drastically reduced in lupus patients’ B cells. We found that the signaling of BCR, TLR9 and reactive oxygen species (ROS) all upregulate the transcription of mouse SMS2 gene, *Sgms2*, in cultured mouse B cells (180).

In addition to B cell activation, the BCR plays an essential role in central B cell tolerance (127). Immature autoreactive B cells that have high-avidity interactions with self-antigen stop their development due to high BCR signaling. These B cells then rearrange and express new light chains to avoid binding with self-antigens. If successful, they will resume B cell development. If self-reactivity remains, they will undergo apoptosis or anergy. In peripheral B cell tolerance, self-antigen-induced BCR signaling, such as the Lyn-SHIP-1-SHP-1 pathway, has an inhibitory effect that prevents the activation of autoreactive B cells including lupus autoreactive B cells (130). In line with these observations, we found that BCR signaling upregulated SMS2 and induced PKCδ-mediated apoptosis in cultured B cells (180), suggesting the involvement of BCR signaling in regulation of the SMS2/PKCδ tolerance pathway.

Given that *Sgms2* transcription in macrophages was strongly activated by the stimulation of TLR9 (192), a pathogen pattern recognition receptor for microbial dsDNA, we studied if stimulation of TLR9 induced SMS2 expression in B cells and compared it to the stimulation of TLR7, a key sensor of single-stranded RNA for triggering antiviral responses. Studies have showed that B cell intrinsic TLR9 and TLR7 play opposing physiologic roles in autoantibody production (193). B cell specific TLR9 deficiency aggravated nephritis in MRL/lpr mice, whereas B cell-specific TLR9 overexpression ameliorated nephritis (194). However, B cell specific TLR7 deficiency significantly relieved disease in B cell specific TLR9 deficient MRL/lpr mice (195). Consistently, a TLR7 gain-of-function mutation causes lupus in humans and mice (196). In agreement with these results, we found that TLR9 stimulation highly upregulated B cell SMS2, but TLR7 stimulation had a minor effect on B cell SMS2 (180). Importantly, upregulation of B cell SMS2 by TLR9 helps clarify why the SMS2/PKCδ tolerance pathway is more specifically activated in anti-dsDNA GC B cells.

Interestingly, we also found that ROS increased SMS2 expression and SMS2-dependent PKCδ nuclear localization in culture B cells (180). We showed that the ROS level was significantly higher in GC LZ B cells compared to GC dark zone (DZ) B cells, which helps explain increased SMS2 expression in LZ B cells. Therefore, observed involvement of BCR, TLR9 and ROS signaling in promoting *Sgms2* transcription supports the SMS2/PKCδ pathway’s tolerance role in the GC. The regulation of B cell SMS2 expression by other unknown signaling pathways cannot be excluded. Future studies are needed to fully understand the downregulation of B cell SMS2 expression in lupus patients. In addition to reduced B cell SMS2 expression, a possible reduction of SMS2 activity in lupus B cells cannot be excluded, as we found that pharmacologic activation of SMS2 restored PKCδ nuclear translocation in the GC B cells of lupus prone NZBWF1 mice (180). Therefore, the studies of SMS2 activity in lupus patients’ B cells are needed as well.

It has been shown that PKCδ nuclear translocation in B cells was blocked by B cell activating factor (BAFF) (182), which is often overexpressed and closely linked with disease activity in lupus patients (197, 198). BAFF is well known for its activity to promote lupus autoimmunity (199); BAFF transgenic mice exhibit a lupus-like phenotype with impaired B cell tolerance (200). Therefore, we postulate that overexpressed BAFF inhibits the SMS2/PKCδ tolerance pathway. In addition to BAFF, other upregulated cytokines known to promote lupus autoimmunity, such as type I interferons (IFNs), may possibly regulate B cell PKCδ nuclear translocation as well. This is because IFNs regulate PKCδ activation (201–203), and IFN-beta can rapidly reverse nuclear translocation of PKCδ in T cells (204). A study of tumor necrosis factor receptor-associated factor 3 (TRAF3) deficient mice also suggested the presence of other unknown inhibitors of PKCδ nuclear translocation in B cells (205). TRAF3 deficient mice exhibit a lupus-like phenotype associated with impaired PKCδ nuclear translocation in B cells, but with an unknown mechanism. Given well-recognized role of upregulated BAFF and IFN signaling in promoting lupus pathogenesis, confirming their inhibitory effects on PKCδ nuclear translocation in GC B cells may provide important insights into impaired GC B cell tolerance in lupus and further justify the SMS2/PKCδ tolerance pathway as a therapeutic target for lupus treatment.

### Restoring the SMS2/PKCδ tolerance pathway with 2OHOA

2-hydroxyoleic acid (2OHOA) is an orally bioavailable SMS activator (206–209), which is being studied in a clinical trial for glioma treatment in Europe. The use of 2OHOA for glioma treatment is based on the finding that reduced SMS2 expression promotes survival of glioma cells (207, 208). A report of its phase I/IIa results confirmed that 2OHOA is safe and nontoxic (206). We therefore repurposed 2OHOA as an activator of the SMS2/PKCδ tolerance pathway for lupus treatment.

How 2OHOA activates SMS is not completely understood. SMS1 and SMS2 are two main isoforms of SMS enzymes in mammalian cells. SMS1 is located on the Golgi membrane, and SMS2 is predominantly situated on the plasma membrane (181). Analysis of both human SMS isoforms’ tertiary structures predicated by deep learning-based folding algorithms suggested that 2OHOA preferentially activates SMS2 (210). This is because the incorporation of 2OHOA into ceramide generates hydroxylated ceramide, which is largely favored in the interaction with SMS2, but not SMS1, and this interaction can lead to increased sphingomyelin and DAG in the plasma membrane. Consistently, we found that 2OHOA upregulated plasma membrane DAG in B cells in a SMS2 dependent manner (180). This is likely due to upregulated SMS2 activity, as 2OHOA did not increase SMS2 expression in B cells. Interestingly, despite most reports that 2OHOA upregulates SMS activity, a study showed that 2OHOA failed to increase SMS activity in certain cancer cells, but reduced cellular phosphatidylcholine instead (211). This result, however, aligns with recently reported new functions of SMS enzymes, namely, the hydrolysis of phosphatidylcholine and phosphatidylethanolamine (212–214). Notably, these two reactions also generate DAG as a by-product. Therefore, SMS2 may utilize different mechanisms to generate DAG. More studies are needed to fully understand how 2OHOA stimulates SMS2 to produce DAG in autoreactive B cells.

In sum, 2OHOA appears to be a potent activator of the SMS2/PKCδ tolerance pathway. In spite of reduced GC B cell SMS2 in female NZBWF1 mice, we reported that pharmacologic activation of the remaining GC B cell SMS2 in female NZBWF1 mice with a half dose of 2OHOA used for glioma treatment in mice was still able to restore PKCδ nuclear translocation in their GC B cells and relieve lupus pathogenesis without decreasing total IgGs or causing other obvious adverse effects (180). As predicted, 2OHOA’s therapeutic effects depended on the SMS2/PKCδ tolerance pathway since 2OHOA could not suppress lupus autoimmunity in SMS2 deficient mice, PKCδ deficient mice, or GC B cell-specific PKCδ deficient mice (180). These results support that the SMS2/PKCδ tolerance pathway can be targeted by 2OHOA to selectively and effectively suppress lupus autoimmunity.

### Restoring the SMS2/PKCδ tolerance pathway with belimumab

Belimumab, a monoclonal antibody that inhibits BAFF, has been approved for lupus treatment by the FDA. However, despite its effective blockade of BAFF, which is frequently overexpressed in lupus patients and promotes lupus autoimmunity (215–217), belimumab is only indicated as an “add-on” therapy with standard of care treatments of lupus because it has moderate efficacy (218–223).

Overexpressed BAFF is thought to dysregulate negative selection of autoreactive B cells (224); studies have showed that overexpressed BAFF rescued the maturation of autoreactive cells from the negative selection at transitional B cell stage (225, 226). In the GC, the role of BAFF in B cell tolerance is poorly understood, but blockade of BAFF signaling significantly reduced GC T follicular helper cells and GC B cells in autoimmune-prone BCMA-deficient B6.Nba2 mice (227). A recent study showed that BAFF promotes GC formation, which was dependent on BR3 expression on both T cells and B cells (228). Consistently, the progression of GC responses was found to be attenuated in BAFF or BAFF-R signaling deficient mice (229). A clinical study of a long-term belimumab treatment suggested a possible involvement of overexpressed BAFF in inhibiting GC B cell tolerance, as treated patients had a modest reduction in the frequency of activated autoreactive B cells, but not inactive autoreactive B cells (230). Along with the fact that overexpressed BAFF promotes lupus autoimmunity (199, 200), it is possible that overexpressed BAFF may inhibit the SMS2/PKCδ tolerance pathway in lupus patients. This hypothetical model provides an explanation of belimumab’s moderate efficacy. Because BAFF blockade by belimumab may not be enough to restore the SMS2/PKCδ tolerance pathway due to significantly reduced SMS2. This view is supported by the observation that in female NZBWF1 mice, which have upregulated serum BAFF levels and reduced GC B cell SMS2 expression (SS and CH, unpublished observations) (231–233), BAFF blockade with BAFF-receptor-Ig could not reverse, but delayed and limited lupus pathogenesis, without a significant reduction of anti-dsDNA antibodies (234–236). Therefore, a possible approach to addressing moderate efficacy of belimumab is to activate SMS2 with 2OHOA, which we reported as able to restore PKCδ nuclear translocation in GC B cells of female NZBWF1 mice (180).

## Summary

The well-recognized adverse effects of current lupus medications indicate an urgent need of novel alternative therapeutic approaches that selectively suppress lupus autoimmunity. As summarized in **Table 1**, long-term efforts have been made to selectively target lupus autoimmunity by reinforcing immune tolerance in various ways. Among them, approaches directly blocking anti-dsDNA antibodies, leveraging Treg-mediated B cell extrinsic tolerance, and reinforcing B cell intrinsic tolerance to lupus autoimmunity by leveraging inhibitory mechanisms with unknown physiological connection to lupus, have been extensively studied but not yet achieved satisfactory clinical effectiveness. In contrast, restoring physiological B cell intrinsic tolerance to SHM-derived autoreactive BCRs has been barely studied despite SHM being identified as the main generator of lupus autoimmunity. This is largely because of poorly understood mechanisms of GC B cell tolerance. The SMS2/PKCδ pathway regulates GC B cell tolerance, as it functions in the GC LZ and its impairments result in a lupus-like phenotype in mice. In addition, evidence suggests that insufficiency in the SMS2/PKCδ pathway is a common defect in lupus patients. To further justify the therapeutic approach to restoring the SMS2/PKCδ pathway for lupus treatment, more studies are needed to demonstrate that this tolerance pathway is a major self-protective mechanism protecting against lupus autoimmunity in humans.

Our earlier discovery showed that restoring the SMS2/PKCδ tolerance pathway could effectively and specifically suppress lupus autoimmunity in NZBWF1 mice, supporting that targeting GC B cell intrinsic tolerance alone is sufficient to relieve lupus pathogenesis in mice. However, lupus disease in humans is well known for its complexity and heterogeneity (2–7), and medications targeting single agents are rarely successful. We believe that the SMS2/PKCδ tolerance pathway can be potentially complemented by the mechanisms of action of other medications to generate synergistic therapeutic effects. For example, concurrent reinforcement of B cell intrinsic and extrinsic tolerance by activation of both SMS2/PKCδ pathway and Tregs may maximize the strength of B cell tolerance to lupus autoimmunity. In addition, combination of the activation of the SMS2/PKCδ pathway with current lupus medications has the potential to enhance therapeutic effectiveness while reducing adverse events by allowing for a reduction in therapeutic doses of current medications. In summary, we believe that restoring patients’ physiological B cell intrinsic tolerance to lupus autoimmunity is feasible and presents a viable and effective strategy to overcome the serious challenge of adverse effects in current medications, therefore representing a new direction for lupus treatment.
